# Complex genetic mechanisms in glaucoma: An overview

**DOI:** 10.4103/0301-4738.73685

**Published:** 2011-01

**Authors:** Kollu N Rao, Srujana Nagireddy, Subhabrata Chakrabarti

**Affiliations:** Kallam Anji Reddy Molecular Genetics Laboratory, Prof. Brien Holden Eye Research Centre, L.V. Prasad Eye Institute, Hyderabad, India

**Keywords:** Glaucoma, genes, mutations, proteins

## Abstract

Glaucomas comprise a group of hereditary optic neuropathies characterized by progressive and irreversible visual field loss and damage to the optic nerve head. It is a complex disease with multiple molecular mechanisms underlying its pathogenesis. Genetic heterogeneity is the hallmark of all glaucomas and multiple chromosomal loci have been linked to the disease, but only a few genes have been characterized, viz. myocilin (*MYOC*), optineurin (*OPTN*), *WDR36* and neurotrophin-4 (*NTF4*) in primary open angle glaucoma (POAG) and *CYP1B1* and *LTBP2* in congenital and developmental glaucomas. Case-control-based association studies on candidate genes involved in different stages of glaucoma pathophysiology have indicated a very limited involvement. The complex mechanisms leading to glaucoma pathogenesis indicate that it could be attributed to multiple genes with varying magnitudes of effect. In this review, we provide an appraisal of the various efforts in unraveling the molecular mystery in glaucoma and also some future directions based on the available scientific knowledge and technological developments.

Glaucoma is a complex disease that comprises a group of heterogeneous optic neuropathies characterized by a progressive degeneration of the optic nerve head and visual field defects.[1] It affects 70 million people and is the second leading cause of blindness worldwide. It is estimated that by the year 2020, this number would rise to around 79.6 million.[1] The prevalence of glaucoma varies widely across the different ethnic groups and is significantly higher in blacks (4.7%) than in the white population (1.3%).[2] In India it is estimated that glaucoma affects 12 million people and causes 12.8% of the total blindness in the country. It is considered to be the third most common cause of blindness with a prevalence ranging from 2.6-4.1%.[3–5]

Elevated intraocular pressure (IOP) is a major risk factor in glaucoma, and experimental elevation of IOP has resulted in glaucoma in animal models.[6] The other common risk factors include age, race, family history, thin cornea, myopia and oxidative stress.[7] Family history of glaucoma is estimated to account for a risk of 1-10 folds among the first-degree relatives of an affected individual.[8]

Glaucomas are categorized into primary and secondary based on their etiology and aqueous humor dynamics.[9] Based on gonioscopy, primary glaucomas are further classified as primary open angle glaucoma (POAG) and primary angle closure glaucoma (PACG). POAG may be associated with or without an elevated IOP and has an adult onset (usually >35 years) or juvenile onset (usually <35 years).[9] Secondary glaucomas are characterized by the involvement of predisposing ocular or systemic diseases such as uveitis, trauma, and diabetes thereby resulting in an alteration of aqueous humor dynamics. These include pseudoexfoliation glaucoma (XFG) and pigmentary glaucoma (PG).[9] The mode of inheritance in adult-onset POAG and PACG is complex in nature. This has limited the identification of large affected families for gene mapping by linkage analysis. On the other hand, the hereditary component in juvenile-onset POAG has facilitated mapping of some candidate loci.[10]

Developmental glaucomas include primary congenital glaucoma (PCG) and glaucoma-associated syndromes (Aniridia and Axenfeld Rieger syndrome).[9] These glaucomas largely follow a Mendelian pattern through autosomal dominant and autosomal recessive modes of inheritance. Thus, co-segregation of candidate gene mutations or the disease-susceptible alleles among the affected subjects in such families are relatively easy to determine.[11]

Among these glaucoma subtypes, primary glaucomas (largely POAG) are the most common form and are attributed to multiple genes with varying magnitudes of effect.[12] Gene mapping in these disorders pose a major challenge but the success of new-generation technologies and high-throughput screening platforms have provided some hope in understanding their underlying molecular mechanisms. Herein, we present an update on the molecular genetics of primary glaucomas, particularly POAG.

## Chromosomal Loci Mapped in POAG

Glaucoma being a complex disorder is attributed to several genes, the majority of which are yet unidentified. Until now, linkage analysis in large affected families has yielded 25 chromosomal loci linked to POAG. Of these, 15 have been designated as *GLC1A* to *GLC1O* by the HUGO genome nomenclature committee (www.gene.ucl.ac.uk/nomenclature) and mutations in only four genes have been identified in POAG. These include myocilin (*MYOC, GLC1A*), optineurin (*OPTN; GLC1E*), WD repeat domain 36 (*WDR36, GLC1G*) and neurotrophin-4 (*NTF4, GLC1O*). Further details are provided in Table 1. Although these genes harbor POAG-associated mutations, they exhibit a high degree of allelic heterogeneity in different populations. The mutation spectra in these genes do not indicate their overall involvement in the disease pathogenesis. The problem of understanding the underlying molecular mechanism is additionally compounded by the variable penetrance and expressivity of these gene mutations. The other loci that were mapped in POAG are yet to be characterized for the disease-association mutations.

**Table 1:** List of candidate loci identified in glaucoma

Chromosomal location	Phenotype	Locus name	Candidate gene	Reference
1q24.3–q25.2	JOAG, Adult onset	*GLC1A*	*MYOC*	Sheffield *et al*.[10]
2cen–q13	Adult onset	*GLC1B*		Stoilova *et al*.[13]
3q21–q24	Adult onset	*GLC1C*		Wirtz *et al*.[14]
8q23	Adult onset	*GLC1D*		Trifan *et al*.[15]
10p15–p14	Adult onset, NTG	*GLC1E*	*OPTN*	Sarfarazi *et al*.[16] Rezaie *et al*.[17]
7q35–q36	Adult onset	*GLC1F*		Wirtz *et al*.[18]
5q22.1	Adult onset	*GLC1G*	*WDR36*	Monemi *et al*.[19]
2p16.3–p15	JOAG, Adult onset	*GLC1H*		Lin *et al*.[20] Suriyapperuma *et al*,[21]
15q11–q13	Adult-onset POAG	*GLC11*		Allingham *et al*.[22]
9q22	JOAG	*GLC1J*		Wiggs *et al*.[23]
20p12	JOAG	*GLC1K*		Wiggs *et al*.[23]
3p21–p22	Adult onset	*GLC1L*		Baird *et al*.[24]
5q22.1–q32	JOAG	*GLC1M*		Pang *et al*[25]
15q22–q24	JOAG	*GLC1N*		Wang *et al*.[26]
19q13.3	POAG and NTG	*GLC1O*	*NTF4*	Ip *et al*.[27] Pasutto *et al*.[28]
2q33.1–q33.3	POAG			Nemesure *et al*.[29]
10p12.33–p13.3	POAG			Nemesure *et al*.[29]
2p14	POAG			Wiggs *et al*.[30]
14q11	POAG			Wiggs *et al*.[30]
14q21–q22	POAG			Wiggs *et al*.[30]
17p13	POAG			Wiggs *et al*.[30]
17q25	POAG			Wiggs *et al*.[30]
19q12–14	POAG			Wiggs *et al*.[30]
1p 32	POAG			Charlesworth *et al*.[31]
10q 22	POAG			Charlesworth *et al*.[31]
2p14	Elevated IOP			Duggal *et al*.[32]
19q12–q14	Elevated IOP			Duggal *et al*.[32]

IOP: Intraocular pressure, POAG: Primary open angle glaucoma, JOAG: Juvenile open angle glaucoma, NTG: Normal tension glaucoma

## Candidate Genes in POAG

### a) Myocilin (MYOC)

The first evidence of linkage in a large German family with juvenile open angle glaucoma (JOAG) was demonstrated by Sheffield *et al*,[10] on Chromosome lq21-q31 (GLC1A). Later *Myocilin/TIGR* (accession numbers: Nucleotide AH006047, Protein NP_000252) on this locus was characterized to be causative for POAG.[33] This gene is also termed as trabecular meshwork-induced glucocorticoid receptor (TIGR) as it overexpresses due to the induction of glucocorticoid to the cells. It consists of three exons that code for an mRNA of about 2.5 kb and encompasses a 57-kDa glycoprotein of 504 amino acids.[34]

Mutations in *MYOC* have been reported from different populations and account for 2-5% of POAG patients worldwide.[35] Around ~72 mutations have been reported till date and the Gln368Stop mutation is the most common mutation observed across multiple populations except the Japanese.[36] In the Indian scenario, the Gln48His was the predominant mutation observed in cases of JOAG, POAG and PCG.[37] Along with the coding region mutations, a few polymorphisms have been identified, of which the -1000C>G was referred to very frequently with variable degrees of association.[38] The probable disease-causing and benign variations in MYOC are available in the *Myocilin* allele-specific phenotype database (http://www.myocilin.com/variants.php).[39]

Genotype-phenotype correlation has been demonstrated with some *MYOC* mutations. It has been observed that individuals with the T377M mutation usually have an onset in their fourth decade whereas those with P370L and Y437H mutations were diagnosed in the first and second decades with severe clinical presentations. The predominant mutation Q368X had an average onset in the fifth and sixth decades[40] and the C433R mutation exhibited an onset between 17 and 58 years and was largely associated with a higher IOP and vertical cup/disc ratio compared to those without this mutation. Other mutations have exhibited variable clinical manifestations.[41]

#### Structure, properties and expression of MYOC

MYOC is a glycoprotein and consists of two major domains: A myosin-like domain near the N terminal region and an olfactomedin-like domain near the C terminal. The N-terminal region of MYOC contains leucine zipper motifs within two coil-coil domains that are important for its interactions with intracellular, extracellular and cell surface proteins, whereas leucine zipper motifs are important in regulating protein function. The olfactomedin-like domain of MYOC has a homology to a family of olfactomedins with a high degree of conservation across species. The presence of >90% glaucoma-associated mutations in this domain indicate its potential functional importance.[40]

In humans the *MYOC* mRNA is expressed in a number of tissues which include both non-ocular and ocular tissues including the trabecular meshwork (TM) that exhibits the highest level of expression, followed by the sclera, ciliary body, choroid, cornea, iris, lamina cribosa, retina, and optic nerve. The non-ocular tissues include mammary gland, small intestine, thymus, prostate, testis, colon, stomach, thyroid, trachea, bone marrow, and brain.[42]

#### Function of MYOC protein

The normal physiological function of MYOC is still unclear but insights from the knockout animal models indicate that the disease-causing MYOC in humans may act by gain of function. *in vitro* studies have shown that MYOC is involved in the cytoskeletal organization and extracellular matrix (ECM) remodulation.[43] Mutations in *MYOC* may not directly affect its expression but it may interfere with protein folding or stability of the folded protein. The misfolded protein may not secrete and accumulate as soluble and insoluble aggregates but may associate with resident proteins of the endoplasmic reticulum (ER). This may lead to the activation of the unfolded protein response finally leading to apoptotic cell death.[44] The higher expression of MYOC in TM cells results in the intracellular accumulation of MYOC aggregates, which is deleterious to TM cells, thereby resulting in deterioration of their function and subsequent elevation of IOP.[44] Studies on the expression of normal and mutant MYOC in cultured ocular and non-ocular cells have suggested that while normal MYOC is secreted from the cultured cells, very little MYOC is secreted from cells expressing different mutations. Thus it can be surmised that glaucoma results either due to insufficient levels of secreted MYOC or compromised TM cell function caused by congestion of the TM secretory pathway.[45] MYOC is also known to be associated with the mitochondrial pathway and it has been shown that overexpression of MYOC carrying P370L mutation results in higher endogenous ROS (reactive oxygen species) production. This further suggests that mutant MYOC may cause mitochondrial defects which may lead to TM cell dysfunction and cell death.[46]

### b) Optineurin (OPTN)

Sarfarazi *et al*.,[16] identified the second gene in the *GLC1E* region (10p15-p14) called optic neuropathy-inducing protein or Optineurin (*OPTN*). Rezaie *et al*.,[17] initially reported mutations in 16.7% families with hereditary POAG of which most of them had low or normal tension glaucoma (NTG). Subsequently, *OPTN* was screened across different populations that revealed few mutations implicated in NTG and POAG [Table 2]. Among these mutations, the E50K located in the putative bZIP motif appears to be most strongly associated with NTG.

**Table 2:** Distribution of *OPTN* mutations across the world

Country	Phenotype	% frequency of mutations	Predominant mutation (%)	Reference
China	POAG	Single family with 6 affected members	K 322E	Xiao *et al*.[51]
China	POAG	1.6	E103D (0.8), H486R(0.8)	Leung *et al*.[52]
Germany	NTG	1.8	A336G (0.9),A377T (0.9)	Weisschuh *et al*.[53]
India	POAG/NTG	4.1 (POAG), 6.0 (NTG)	M98K (4.1 (POAG), 6.0 (NTG))	Sripriya *et al*.[54]
India	POAG	3	R545Q (3)	Mukhopadhyay *et al*.[55]
Iowa and Japan	POAG	0.2	E50K (0.1),E142P (0.1)	Alward *et al*.[56]
Japan	POAG/NTG	1.1 (POAG) 1.5 (NTG)	H26D (1.1),R545Q(1.5)	Fuse *et al*.[57]
Japan	POAG	0.5	H26D (0.5)	Funayama *et al*.[58]
UK	NTG	1.5	E50K(1.5)	Aung *et al*.[59]
USA	POAG/NTG	16.7	E50K(13.5)	Rezaie *et al*.[17]

POAG: Primary open angle glaucoma, NTG: Normal tension glaucoma, UK: United Kingdom, USA: United states of America

#### Structure and expression of OPTN

*OPTN* contains three non-coding exons in 5’-untranslated region (UTR) and 13 exons that code for 577 amino acids. Alternative splicing in 5’-UTR generates at least three different isoforms, but all have same reading frame (gene accession no: AF420371 to AF420373). The structure of OPTN reveals several motifs, including one bZIP motif, two leucine zippers, coiled-coil motifs and a C-terminal C_2_H_2_-type zinc finger domain. OPTN is expressed in both non-ocular and ocular tissues that include the TM, non-pigmented ciliary epithelium, heart, brain, placenta, skeletal muscle, and kidney.[17]

#### Functions of OPTN

OPTN serves many cellular functions based on its interaction with a variety of proteins such as Rab8, Huntingtin, Myosin VI, RIP, transcription factor IIIA, metabotropic glutamate receptor and TBK1. Based on its interaction with Myosin VI, its role has been proposed in vesicular trafficking between the golgi and plasma membrane.[47] Overexpression of OPTN protects the cells from H_2_O_2_-induced cell death by inhibiting release of Cytochrome C from mitochondria. The common mutation E50K selectively induces the death of retinal ganglion cells (RGCs) due to TNF*α*-induced death of RGC.[48] Recently, it has been shown that OPTN negatively regulates TNF*α*-induced NFκB activation although the exact mechanisms by which these cytokines activate *OPTN* gene expression are yet unknown.[49] OPTN also plays an important role in the regulation of many genes which include MYOC although the mechanism involved is yet to be elucidated.[50]

### c) WDR36

Based on a genomewide scan, Monemi *et al*., (2005) characterized the *WDR36* located on *GLC1G* locus (5q22.1) to be involved in POAG. They identified four mutations in WDR36 among 17 unrelated POAG subjects, 11 with high-pressure and six with low-pressure glaucoma.[19] The mutations were absent in 200 normal control chromosomes and their residues were conserved between WDR36 orthologs in mouse, rat, dog, chimpanzee and humans. Specific ocular expressions and the observed mutations were consistent with a role for *WDR36* in the etiology of both high and low-pressure glaucoma. Analysis of *WDR36* sequence revealed that several sequence alterations were exclusive to POAG patients and encoded predicted amino acid substitutions in conserved residues.[19] However, subsequent reports have revealed that the *WDR36* may not be directly involved with POAG and may simply act as a modifier gene [Table 3].

**Table 3:** Distribution of *WDR36* mutations across the world

Country	Phenotype	% Frequency of mutations	Predominant mutation (%)	Reference
China	POAG	1.8	I713V (1.8)	Fan *et al*.[60]
Germany	POAG/NTG	1.7	P31T, Y97C, D126N, T403A, H411Y, H411L, P487R	Pasutto *et al*.[61]
Germany	NTG	9.8	A449T, D33E, A163V, H212P (1.7 each)	Weisschuh *et al*.[62]
Japan	POAG	0.7	S664L (0.7)	Miyazawa *et al*.[63]
USA	POAG	6.9	D658G (3.85)	Monemi *et al*.[19]
USA	POAG	11	M671V(3.3)	Hauser *et al*.[64]

NTG: Normal tension glaucoma, POAG: Primary open angle glaucoma

#### Structure and expression of WDR36

The gene spans about 38.3-kb genomic region and contains 23 exons expressed predominantly as two transcripts (5.9kb and 2.5kb). The full-length of this protein contains 951 aa harboring four conserved domains: (a) nine WD 40 repeat domain; (b) Utp21 domain;(c) AMP-dependent synthetase and ligase domain, and (d) cytochrome cd1-nitrite reductase-like domain. WDR36 is widely expressed in several ocular and non-ocular tissues.

#### Functions of WDR36

Skarie *et al*., (2008) studied the functional role of WDR36 in zebra fish and found that it is essential for nucleolar processing of 18s rRNA and is thus required for the biogenesis of ribosomes.[65] In zebrafish, loss of WDR36 function resulted in reduced levels of 18s rRNA and also in ocular dysmorphology leading to activation of p53 stress response pathway. This may indicate the possible role of WDR36 sequence variants in POAG pathogenesis. Since variations in p53 are also known to be involved in POAG, co-inheritance of both *P53* and *WDR36* variations may thus be involved in the disease progression.[65]

### d) CYP1B1

The first genetic locus (*GLC3A*) for PCG was mapped by Sarfarazi and co-workers in 1995 based on their study of 17 Turkish families comprising 113 individuals that included 79 offsprings, of which 40 were affected with PCG.[11] Stoilov *et al*., (1997) identified *CYP1B1* as a candidate gene at this locus for PCG[66] and subsequently pathogenic mutations in *CYP1B1* have been identified in PCG with varying frequencies from 20-100% across different populations.[67–72]

*CYP1B1* has also been implicated in juvenile and adult onset forms of glaucoma, in various ethnic groups worldwide. Initially, Vincent *et al*., (2002) showed the involvement of *CYP1B1* and *MYOC* in POAG through a digenic mechanism in a family of East Indian (Guyanese) origin. Based on these observations, it was suggested that PCG and JOAG are allelic variants of *CYP1B1*. It was also hypothesized that *CYP1B1* and *MYOC* might act through a common biochemical pathway with *CYP1B1* acting as a modifier for *MYOC*.[73] Later the association of *CYP1B1* was reported across different populations ranging from 2.2%-23.3% with JOAG, POAG and PACG [Table 4].

**Table 4:** *CYP1B1* in adult-onset *POAG* worldwidea

Country	Phenotype	% frequency of mutations	Predominant mutation (%)	Reference
Canada	JOAG	3 (5)	R368H, g.1546dup10, L345F (1.6 each)	Vincent *et al*.[73]
France	POAG	11 (4.6))	A443G (1.3)	Melki *et al*.[74]
	POAG	3 (2.2)		
Germany	JOAG	3(8.5)	Y81N (1.5)	Pasutto *et al*.[75]
	NTG	1(4.1)		
India	POAG	9 (4.5)	S515L (2)	Acharya *et al*.[76]
India	POAG	27 (10.8)	R368H (4.0)	Kumar *et al*.[77]
India	POAG	18 (17.3)	R368H (5.8)	Chakrabarti *et al*.[78]
	JOAG	7 (23.3)	G61E (3.3)	
	PACG	10 (11.1)	R368H (5.6)	
Iran	POAG	7 (11.1)	R368H (4.7)	Suri *et al*.[79]
Spain	POAG	9 (10.9)	Y81N (3.6)	Lopez-Garrido *et al*.[80]

JOAG: Junvenile open angle glaucoma, NTG: Normal tension glaucoma, POAG: Primary open angle glaucoma, Figures given in parentheses are in percentage

#### Structure and expression of CYP1B1

CYP1B1 encodes a 543 amino acid dioxin inducible member of the cytochrome p450 gene superfamily and consists of three exons that span 8.5 kb of genomic DNA.[73] CYP1B1 is known to express both in ocular and non-ocular tissues. Animal models have shown that CYP1B1 deficiency leads to abnormality in the ocular drainage structures and TM that are similar to those observed in human PCG.

#### Functions of CYP1B1

CYP1B1 is involved in the metabolism of steroids, retinol, retinal, arachidonate and melatonin. Although the exact role of CYP1B1 is unknown, their involvement in metabolizing these steroids may contribute to the regulation of IOP.[81]

### e) NTF4

*NTF4* gene is located on Chromosome 19q13.33 and is translated as pre-pro-neurotrophin and cleaved to release the mature active protein. Lambert *et al*., (2001) have shown that cells within the lamina cribrosa (LC) express neurotrophins [NTs] and trk receptors. These NTs play an important role in neuronal development, survival and differentiation.[82] NTs constitute a family of polypeptide growth factors that promote development, survival and differentiation of neurons. Animal models have shown that elevated IOP, ischemia impairs the neurotrophins’ signaling thereby it leads RGC death.[83] A recent report by Pasutto *et al*., (2009) has shown that mutations in neurotrophin Factor 4 (NTF4) impairs the neurotrophins’ signaling in POAG. Screening of NTF4 revealed 1.7% of POAG patients of European origin carrying mutations in NTF4. Molecular modeling and *in vitro* studies have shown that the mutations reported in this study reduce the binding affinity of NTF4 to its target receptor TrkB thereby reducing the function of neurotrophins.[28] However, later studies have demonstrated the lack of involvement of NTF4 in Indian and Caucasian populations.[84,85]

## Genome-Wide Association Studies

Glaucoma being a complex disease, linkage studies were not very successful in identifying the genes responsible for raised IOP and RGC death. This was further compounded by the late onset of symptoms and the unavailability of large affected families. Genome-wide association studies (GWAS) represent a powerful approach for gene mapping in large cohorts using high-density markers like single nucleotide polymorphisms (SNPs) on microarray platforms. These have revealed several loci associated with several complex diseases like diabetes, rheumatoid arthritis and age-related macular degeneration (AMD).[86,87]

Based on this approach, Thorleifsson *et al*., (2007) identified three SNPs (rs1048661, rs3825942 and rs2165241) in *lysyl oxidase-like protein 1 (LOXL1)* gene on Chromosome 15q22 that were significantly associated with XFS/XFG.[88] Due to the association of POAG and XFS, further analysis of these SNPs in POAG in other populations revealed no significant association in POAG, PACG and PG indicating their exclusive involvement with XFS/XFG only.[89]

A recent study by Nakano *et al*., (2009) demonstrated significant association of six SNPs located on three different chromosomal loci, viz. 1 (*ZP4*), 10 (*PLXDC2*) and 12 (DKFZ*p*762A217) in a Japanese population with both high and low-pressure glaucomas. All these SNPs exhibited significant association with combined *P* values ranging from 1.0X10^-5^to 9.0X^10-5^along with an odds ratio (OR) of 1.33 - 1.49.[90] Replication of these SNPs in an Indian cohort with POAG and PACG indicated that these were not associated with IOP-related glaucomas.[91] The failure of replication may be due to inclusion of cases with high IOP unlike mixture of both high and low-pressure glaucomas in the Japanese cohort.

By using linkage and SNP mapping, Jiao *et al*., (2009) have identified a locus on Chromosome 2 associated with POAG.[92] The region was characterized on Chromosome 2p by performing linkage analyses in 146 multiplex families from Barbados Family Study of Glaucoma (BFSG). Case-control analysis on independent groups from BFSG participants identified a strong association with rs12994401 and POAG. This region overlapped with previous linkage studies in Chinese and African families, indicating that this locus could be a significant cause of glaucoma in the Chinese and Europeans.[20,21]

## Candidate Gene- Based Association Studies

Association studies have suggested many genes in single studies while a few of them have been investigated in multiple studies with conflicting results. The variations in association could be due to racial differences, sample size, poorly characterized controls, and clinical heterogeneity between different populations. The POAG-associated genes include *ANP, APOE, OPA1, P53, GST, Interleukins, and TNFα*. The role of these genes in the etiology of POAG is still controversial [Table 5].

**Table 5:** Different candidate genes associated with glaucoma

Chromosomal location	Gene name	Phenotype	Country	Reference
1p36.2	*ANP*	POAG	Australia	93
		POAG	Australia	94
		NTG	Korea	95
Xq22-q23	*AGTR2*	POAG	Japan	96
		NTG		
17q23.3	*ACE*	POAG	Turkey	97
6p21.2	*P21*	POAG	China	98
9q32-q33	*TLR4*	NTG	Japan	99
19q13.2 and q13.3	*XRCC1* and *XPD*	POAG	Turkey	100
7q21.3-q22	*PAI1*	POAG	Austria	101
20q11.2-q13.1	*MMP9*	PACG	China	102
		PACG	Singapore	103
11q23	*MFRP*	PACG	China	104
5q31.1-q31.2	*Hsp70*	POAG	Japan	105
14q32.1	*CYP46A1*	POAG	France	106
5q32-q34	*B2AR*	POAG	Turkey	107
6p21.3	*TNF alpha*	POAG	China	108
		POAG	Austria	109
		POAG	Japan	110
17p13.1	*P53*	POAG	UK	111
		POAG	India	112
		HTG, NTG	Tasmania	113
		POAG	Brazil	114
		POAG	China	115
		POAG, NTG, OHT	Japan	116
		POAG	Turkey	117
3q28-q29	*OPA1*	NTG	UK	118
		NTG and HTG	Japan	119
		NTG	UK	120
		NTG	Korea	121
		NTG and POAG	West Indies	122
1p36.3	*MTHFR*	POAG	Germany	123
		POAG and NTG	Japan	124
		PACG and POAG	Pakistan	125
		NTG	Korea	126
2q14		POAG	China	127
2q14	*IL1β*	POAG	China	128
	*IL-1β(-511) and IL-iβ*	NTG	China	129
	*(+3953)*	POAG, NTG and PACG	China	130
	*IL1A (-889C/T), IL1B*			
	*(+3953C/T), and IL1B*			
	*(-511C/T)*			
1p13.3 (GSTM1)	*GSTM1*	POAG	Estonia	131
22q11.2 (GSTT1)	*GSTM1andT1*	POAG and PACG	Saudi Arabia	132
	*GSTM1andT1*	POAG	Turkey	133
	*GSTM1*	POAG	Swedon	134
		POAG	France	135
19q13.2	*APOE*	NTG	Tasmania	136
		NTG	UK	137
		POAG	Japan	138
		POAG and PACG	Saudi Arabia	139
		POAG	UK	140
11p15.5	*IGF2*	POAG	China	141

POAG: Primary open angle glaucoma, NTG: Normal tension glaucoma, HTG: High tension glaucoma, PACG: Primary angle closure glaucoma

## Interaction of Genes

Glaucoma is a complex disorder in which a single gene may not contribute to disease progression. It has been demonstrated that Apolipoprotein E promoter SNPs previously associated with Alzheimer’s disease may also modify POAG phenotype. *APOE* (-219G) is associated with increased optic nerve damage. The interaction between *APOE* (−491T) and an *SNP* in the *MYOC* promoter, *MYOC* (−1000G) is associated with increased IOP and with limited effectiveness of IOP-lowering treatments indicating that *APOE* is modifier gene for the *MYOC*.[135] The interaction was also observed between *TNF-α* -863A/C and *OPTN* 603A/T (or met98Lys) and the carriers of *TNF-α*/-863A with OPTN/603A (or Lys98) had significantly worse (*P* = 0.026) visual field scores than those only with *OPTN*/603A (or Lys98).[110] This interaction was consistent at the molecular level by inducing expression of OPTN by TNF*α* through NFκB pathway.[48] The interaction between *MYOC*, *OPTN* along with *APOE* has been suggested to contribute towards the progression of POAG. Common polymorphisms in *MYOC, OPTN* and *APOE* were identified that might interactively contribute to POAG.[50] Some studies also observed interaction between *Noelin 2 (OLFM2*/317A) and *OPTN (OPTN*/412A) and the *OLFM2*/1281T and *OPTN*/412A SNPs with OAG with elevated IOP.[142]

## Gene Expression Studies

The TM tissue plays a key role in the regulation of IOP, and altered TM morphology and functions have been observed in POAG. The mechanisms of action in the TM as well as RGC and astrocytes are dependent on the expression of several genes. Gene expression analysis provides the identification of mechanisms which could be involved in the pathophysiology of POAG. Over the past few years several groups across the world have performed expression studies on TM, RGC and astrocytes that revealed several genes in these tissues. These findings support the existence of numerous regulatory mechanisms in the TM as well as in the optic nerve head (ONH). These genes are involved in the expression of ECM and its remodeling cellular metabolism, cell transport and cell defense.[2,143]

## Factors Involved in The Elevation of IOP

Elevated IOP is the most important risk factor in POAG and all current treatments for glaucoma involve the lowering of IOP. Despite its importance in clinical practice, knowledge of IOP regulation in the human eye is limited. Cells which are present in the outflow pathway play an important role in IOP regulation. Recent reports have shown that there are several factors which are involved in maintaining the TM physiology thereby regulating IOP. Some of the important factors involved in raised IOP are age, abnormal function of genes, alterations of ECM and oxidative stress. Age plays an important role in the regulation of IOP because decreased TM cellularity with age could alter the synthetic and catabolic control of the extracellular environment. As the TM cellularity decreases with age their phagocytic activity is lost and leads to accumulation of several toxic molecules within the drainage channels thereby obstructing outflow.[144] Studies have also shown that genetic factors play an important role in the regulation of IOP. Several *in vitro* and *in vivo* studies have shown that mutations in the MYOC are a known cause of the abnormal function of TM causing elevation of IOP.[44] Animal models have shown that *BAX, SPARC, bestrophin-2 (Best2*) and *aquaporin* deficiency limits elevation of IOP and these results indicate that multiple pathways regulate IOP.[145–148] Several other molecules like endothelial leucocyte adhesion molecule-1 (ELAM), endothelins (ET), nitric oxide (NO) and modification of ECM molecules have been altered under oxidative stress and these are involved in raising of IOP.[149] ECM components of the TM play an important role in the regulation of IOP and several ECM molecules have been upregulated in TM of POAG patients. Along with oxidative stress, transforming growth factor beta (TGFβ) plays an important role in modulation of ECM molecules in TM. TGFβ2 is the predominant isoform in the eye and several groups have reported higher levels in the aqueous of POAG patients. Recently, adenoviral gene transfer of active human TGFβ2 has been shown to elevate IOP and reduces outflow facility in rodent eyes, indicating that increased levels of TGFβ2 play a major role in the elevation of IOP in POAG.[150]

## Mechanism of RGC Death

Progressive loss of optic nerve axons and RGCs result in characteristic optic nerve atrophy and visual field defects in glaucoma patients. A number of hypotheses have been proposed to trigger ganglion cell injury and death in glaucoma. These include compromise to blood flow at the optic nerve, mechanical compression due to raised IOP, loss of neurotrophic factors, autoimmune mechanisms, nitric oxide-induced injuries to the optic nerve and glutamate excitotoxicity. A combination of these factors may be involved in causing glaucomatous RGC loss. Increased IOP is a major risk factor and is involved in the formation of mechanical stress on the ONH thereby inducing glaucomatous cell death. The ONH consists of axons which are projected from RGC, which exit the eye through the lamina cribrosa (LC), a collagenous structure with sieve-like openings. Elevated IOP causes mechanical stress on the LC which leads to decreased axonal transport which causes deprivation of neurotrophic factors. Animal models have shown that acute IOP elevation causes blockage of brain-derived neurotrophic factor (BDNF) transport and may contribute to neuronal death. Supplementation of neurotrophins transiently protect the retina from pressure-induced ischemic injury indicating that neurotrophin deprivation is involved in RGC death.[83]

Tissue hypoxia in the ONH and/or retina is thought to develop secondary to or independent from the elevated IOP in glaucomatous eyes and has been proposed to be associated with pathogenic mechanisms underlying optic nerve degeneration in glaucoma. Considerable evidence suggests that tissue hypoxia in the retina may adversely affect the survival of retinal ganglion cells by inducing apoptosis.[151] Brief preconditioning hypoxia induces HIF-1*α* expression in the retina, which accompanies the expression of adaptive proteins and provides resistance to cell death; however, exposure to hypoxia for a longer period initiates the cell death program.[151]

The eye is an organ that is predisposed to great levels of oxidative stress.[152] Oxidative stress evident in glaucomatous tissues is an important factor for the loss of neurons during glaucomatous neurodegeneration. RGC death induced by glaucomatous stimuli involves receptor-mediated caspase cascade, and mitochondria-mediated caspase-dependent and caspase-independent components of cell death cascade. TNF-*α* and hypoxia are two different stimuli known to preferentially trigger the receptor-mediated or mitochondria-mediated cell death pathways, respectively. Following oxidative modifications of retinal proteins in glaucomatous eyes, reduced ability of cells to cope with the glaucomatous tissue stress may result in impaired cellular homeostasis eventually contributing to neurodegeneration.[153–156]

Nitric oxide (NO) is another important mediator of glial cell-mediated apoptosis in neuronal cells. Under normal physiological conditions NO plays an important role in performing several physiological functions, including regulation of vascular tone, neurotransmitter release and synaptic plasticity. NO can be synthesized by three enzymes (NOS-1, NOS-2, and NOS-3) and elevated levels of NO have been observed in the AqH and genetic association of iNOS polymorphisms have been reported in glaucoma patients.[157]

Neurotrophins, particularly BDNF, are known to influence RGC survival *in vitro*, both during retinal development and after lesioning.[158] Loss of physiological neurotrophin levels, particularly BDNF is consistent with known events in the clinical and pathological aspects of glaucoma. Overexpression of BDNF delays RGC death in experimental glaucoma.

## Immune Response

Increasing evidence from both *in vivo* and *in vitro* studies over the past few years strongly supports the presence of the immune system in glaucoma pathogenesis. Recent studies have shown increased expression of several autoantibodies against many optic nerve and retinal proteins. These antibodies include hsp60, hsp27, alpha crystallins, vimentin and HSP70. The direct application of Abs against these proteins at similar concentration found to induce RGC death in *in vivo* and *in vitro* conditions. Recent reports have also shown that complement cascades have been implicated in glaucomatous neurodegeneration. IOP also modulates the immune system by inducing several complement components like C3, C1q and C3r. Interestingly, it has been shown that complement factor H, a common regulator of the complement system is down regulated, while several other complements components are up-regulated, indicating an abnormal activation of complement system.[159,160]

## The Future

Glaucoma is a complex disease attributed to multiple genes with varying magnitudes of effect. Newer methods of gene mapping involving GWAS have revealed some interesting results over the last few years. However, many of these studies could not be replicated due to differences in phenotyping, other screening modalities and improper study designs. Several genes implicated through functional studies could not be associated in the genetic studies. Since the effect sizes of these genes would vary, there would be a need for precise characterization at the molecular level using multiple approaches. Whole genome sequencing would be an ideal choice for understanding the unknown genes involved in glaucoma pathogenesis. This would need to be supplemented with functional studies and further validation through proteomic approaches, animal models and replications in different ethnic populations. A collective effort including a multi-disciplinary approach involving the expertise of other branches of science is a must for unraveling the mystery and the underlying molecular mechanisms in glaucoma.
